# Deletion of CRH From GABAergic Forebrain Neurons Promotes Stress Resilience and Dampens Stress-Induced Changes in Neuronal Activity

**DOI:** 10.3389/fnins.2019.00986

**Published:** 2019-09-20

**Authors:** Nina Dedic, Claudia Kühne, Karina S. Gomes, Jakob Hartmann, Kerry J. Ressler, Mathias V. Schmidt, Jan M. Deussing

**Affiliations:** ^1^Molecular Neurogenetics, Max Planck Institute of Psychiatry, Munich, Germany; ^2^Department of Psychiatry, Harvard Medical School and McLean Hospital, Belmont, MA, United States; ^3^Laboratory of Neuropsychopharmacology, Paulista State University, Araraquara, Brazil; ^4^Stress Resilience, Max Planck Institute of Psychiatry, Munich, Germany

**Keywords:** corticotropin-releasing hormone (CRH), stress, anxiety, resilience, GABAergic circuits, corticosterone, HPA (hypothalamic-pituitary-adrenal) axis

## Abstract

Dysregulation of the corticotropin-releasing hormone (CRH) system has been implicated in stress-related psychopathologies such as depression and anxiety. Although most studies have linked CRH/CRH receptor 1 signaling to aversive, stress-like behavior, recent work has revealed a crucial role for distinct CRH circuits in maintaining positive emotional valence and appetitive responses under baseline conditions. Here we addressed whether deletion of CRH, specifically from GABAergic forebrain neurons (*Crh^*CKO*–GABA^* mice) differentially affects general behavior under baseline and chronic stress conditions. Expression mapping in *Crh^*CKO–GABA*^* mice revealed absence of *Crh* in GABAergic neurons of the cortex and limbic regions including the hippocampus, central nucleus of the amygdala and the bed nucleus of the stria terminals, but not in the paraventricular nucleus of hypothalamus. Consequently, conditional CRH knockout animals exhibited no alterations in circadian and stress-induced corticosterone release compared to controls. Under baseline conditions, absence of *Crh* from forebrain GABAergic neurons resulted in social interaction deficits but had no effect on other behavioral measures including locomotion, anxiety, immobility in the forced swim test, acoustic startle response and fear conditioning. Interestingly, following exposure to chronic social defeat stress, *Crh*^*CKO–GABA*^ mice displayed a resilient phenotype, which was accompanied by a dampened, stress-induced expression of immediate early genes *c-fos* and *zif268* in several brain regions. Collectively our data reveals the requirement of GABAergic CRH circuits in maintaining appropriate social behavior in naïve animals and further supports the ability of CRH to promote divergent behavioral states under baseline and severe stress conditions.

## Introduction

Since its discovery in 1981 by Wylie Vale and colleagues (Vale et al., 1981), the neuropeptide corticotropin-releasing hormone (CRH) has become known as the key orchestrator of the neuroendocrine, autonomic and behavioral responses to stress (Deussing, 2013; Janssen and Kozicz, 2013; Dedic et al., 2017; Deussing and Chen, 2018). CRH is densely expressed in the paraventricular nucleus of the hypothalamus (PVN) from where it regulates hypothalamic-pituitary-adrenal (HPA) axis activity and consequently the circadian and stress-mediated release of glucocorticoids. Together with its high affinity type 1 receptor (CRHR1), CRH is also involved in modulating behavioral adaptations to stress, which can be attributed to their wide distribution within the mammalian brain including the cortex, key limbic structures and midbrain monoaminergic nuclei (Swanson et al., 1983; Van Pett et al., 2000; Rodaros et al., 2007; Refojo et al., 2011; Dedic et al., 2018a).

We and others have shown that CRHR1 is expressed in forebrain glutamatergic and GABAergic neurons, dopaminergic neurons of the ventral tegmental area (VTA) as well as a limited subset of serotonergic neurons of the dorsal raphe nucleus (Van Pett et al., 2000; Refojo et al., 2011). In contrast, cortical and limbic CRH expression is largely confined to GABAergic neurons with the exception of the piriform cortex (Pir) and PVN, where CRH is found in glutamatergic cells (Chen et al., 2001; Kubota et al., 2011; Dabrowska et al., 2013; Kubota, 2013; Dedic et al., 2018a; Gunn et al., 2019). Although it is well established that CRH/CRHR1 signaling mediates aversive responses, including anxiety and depression-like behaviors, several recent studies have challenged this viewpoint by revealing anxiolytic and appetitive properties of specific CRH/CRHR1 circuits. Genetic dissection of CRHR1-expressing cell populations demonstrated a bidirectional role for the receptor in anxiety, suggesting that glutamatergic and dopaminergic systems mediate anxiogenic and anxiolytic effects of CRHR1, and might function in a concerted but antagonist manner to keep emotional responses to stressful situations in balance (Refojo et al., 2011). More recently, our work extended these findings by identifying the source of the “anxiolytic” CRH neurons. These represent a distinct subpopulation of GABAergic, long-range projecting neurons in the extended amygdala that target CRHR1 on dopaminergic VTA neurons to positively modulate emotional behavior by regulating dopaminergic neurotransmission (Dedic et al., 2018a). Importantly, these findings were obtained from naïve animals, which poses the question as to whether specific CRH circuits might modulate different behaviors under baseline and stressful conditions. In fact, Lemos and colleagues demonstrated that CRH acts in the nucleus accumbens (NAc) to increase dopamine release and promote appetitive behavior in mice; an effect which was lost following repeated stress exposure, suggesting that CRH differentially affects the reward circuitry under basal and stress conditions (Lemos et al., 2012). However, whether severe stress is able to switch CRH action from positive to negative in the context of anxiety, social and/or depression-like behavior, remains largely unexplored. In order to address this question, we assessed whether conditional deletion of CRH from forebrain GABAergic neurons (*Crh*^*CKO–GABA*^ mice) would differently affect anxiety, social behavioral and cognitive parameters under baseline and chronic stress conditions. In contrast to utilizing full CRH knockout mice, this approach enabled the dissection of GABAergic CRH circuits in a genetically defined manner, without altering CRH levels in the PVN and thus not affecting peripheral glucocorticoid release. Our results demonstrate that absence of CRH from forebrain GABAergic neurons increases social avoidance, thus highlighting CRH’s capacity to positively regulate specific behaviors under physiological conditions. In contrast, *Crh*^*CKO–GABA*^ mice exhibited resilience to chronic social defeat stress (CSDS) and showed a decrease in stress-induced neuronal activation in multiple forebrain regions. Collectively, our data demonstrates that CRH can promote divergent effects on specific emotional states under physiological and chronic stress conditions.

## Materials and Methods

### Animals

Adult, male mice were used in all experiments. *Crh*^*CKO–GABA*^ mice were obtained by breeding the recently generated *Crh*^*flox*^ mice (Dedic et al., 2018a) to *Dlx5/6-Cre* driver mice (Monory et al., 2006) to obtain *Crh*^*Ctrl*^ (*Crh*^*lox/lox*^) and *Crh*^*CKO–GABA*^ (*Crh^*lox/lox*^:Dlx5/6-Cre*) mice. Mice were of a mixed 129S2/Sv × C57BL/6J genetic background. All animals were group housed (maximum 4 mice per cage) under standard laboratory conditions (22 ± 1°C, 55 ± 5% humidity) and were maintained on a 12:12 h light–dark cycle (lights on from 07:00 to 19:00 h), with food and water provided *ad libitum*. Behavioral testing was conducted between 8:30 a.m. and 12:30 p.m. during the light cycle. Mice were single housed 1 week before behavioral testing or hormone assessment. For the assessment of initial baseline behavior, the following behavioral tests were performed in one cohort of animals in the following order: open field test, elevated plus-maze, dark/light box test and forced swim test (FST). The acoustic startle response and fear conditioning were assessed in a second cohort of animals. A third cohort of mice was used for the CSDS experiment. All experiments were conducted in accordance with the Guide for the Care and Use of Laboratory Animals of the Government of Upper Bavaria, Germany.

### *In situ* Hybridization (ISH)

ISH was performed as previously described (Refojo et al., 2011). Mice were killed with an overdose of isoflurane (Floren, Abbott) and decapitated immediately after. The brains were carefully removed and immediately shock-frozen on dry ice. Brains were sectioned coronally at 20 μm using a cryostat (Microm, Walldorf, Germany). The sections were thaw-mounted onto SuperFrost slides, dried, and kept at −80°C. The following riboprobes were used: *Crh* (3′UTR): bp 2108–2370 of AY128673; *c-fos*: bp 608–978 of NM_010234; *zif268*: bp 245–786 of NM_00791. Images were analyzed with Adobe Photoshop CS2 and Adobe Illustrator CS2.

### Corticosterone Measurements

To determine basal plasma corticosterone hormone levels, blood sampling was performed in the early morning (08:30–09:30 h) and afternoon (04:30–05:30 h) by collecting blood from the tail vein. Samples were collected in 1.5 ml EDTA-coated microcentrifuge tubes (Kabe Labortechnik, Germany). All blood samples were kept on ice and later centrifuged at 8000 rpm at 4°C for 15 min. Plasma was transferred to new labeled microcentrifuge tubes and stored at −20°C until further processing. Plasma corticosterone concentrations were measured using a commercially available RIA kit (MP Biomedicals, Eschwege, Germany) according to the manufacture’s manual.

### Open Field (OF) Test

The OF test was used to characterize locomotor activity in a novel environment. Testing was performed in an open field arena (50 × 50 × 50 cm) dimly illuminated (about 15 lux) in order to minimize anxiety effects on locomotion. All mice were placed into a corner of the apparatus at the beginning of the trial. The distance traveled, and time spent in the outer and inner zones was assessed with the ANY-maze software (4.20, Stoelting).

### Dark/Light (DaLi) Box Test

The DaLi box test was used to assess anxiety-related behavior and performed in a rectangular apparatus (15 × 20 × 25 cm) consisting of an aversive brightly lit compartment (700 lux) and a more protective dark compartment (5 lux). At the start of the test, all mice were placed in the dark compartment and were allowed to freely explore the apparatus for 5 min. Lit zone entries were counted if at least the two front paws and half of the animal’s body were inside the lit compartment. Automatic tracking was employed using the ANY-maze software (4.20, Stoelting).

### Elevated Plus Maze (EPM) Test

In addition to the DaLi, the EPM was used to assess anxiety-related behavior. The apparatus consisted of a plus-shaped platform with four intersecting arms, elevated 37 cm above the floor. Two opposing open (30 × 5 cm) and closed (30 × 5 × 15 cm) arms were connected by a central zone (5 × 5 cm). Animals were placed in the center of the apparatus facing the closed arm and were allowed to freely explore the maze for 5 min. Automatic tracking was employed using the ANY-maze software (4.20; Stoelting). Percent open arm time was calculated as follows: open arm time (%) = open arm time/(open arm time + closed arm time).

### Social Avoidance Test

The two-trial social avoidance test was modified from Berton et al. (2006), Golden et al. (2011). In the first trial, each experimental mouse was introduced into the open field arena for 2.5 min containing an empty wire mash cage, placed at one side of the apparatus (marked as the interaction zone). During the second 2.5 min trial, test animals were confronted with an unfamiliar male CD1 mouse, which had previously been introduced into the wire mash cage. The ratio between the time in the interaction zone of the non-target trial and the time in the interaction zone of the target trial was calculated.

### Forced Swim Test (FST)

The FST represents a well-established antidepressant-screening paradigm (Porsolt et al., 1977). Animals were carefully placed into a 2 l glass beaker (diameter: 13 cm, height: 24 cm) filled with tap water (22 ± 1°C) to a height of 15 cm, so that the mouse could not touch the bottom with its hind paws or tail. Testing duration was 6 min. The time spent immobile was scored by an experienced observer, blind to genotype or condition of the animals.

### Acoustic Startle Response (ASR)

The ASR was modified from Golub et al. (2009). Mice were introduced into a non-restrictive plexiglas cylinder, which was mounted to a plastic platform located in a sound attenuated chamber (SR-LAB, San Diego Instruments SDI, San Diego, CA, United States). This set-up quantified changes in the conductance as a response to varying acoustic stimuli, which are then detected by a piezoelectric sensor located underneath each cylinder. The background noise was set to 50 dB. After an acclimatization period of 5 min, the mice were subjected to white noise bursts of varying intensities (75, 90, 105, and 115 dB) in a random order. The data are represented as mean peak startle amplitude in mV ± SEM in response to 136 randomized trials of the mentioned intensities including background noise measurements.

### Fear Conditioning

Contextual and cued fear conditioning was performed in conditioning chambers (ENV-307A, MED Associates Inc.) as previously described (Dedic et al., 2018a). Foot shock (FS) delivery and context-dependent fear memory were assessed in a cube-shaped chamber with metal grid floors, which was thoroughly cleaned and sprayed with 70% ethanol before the animals were introduced (shock context). A neutral context consisting of a Plexiglas cylinder with bedding was used to investigate cued (tone-dependent) fear memory; it was cleaned and sprayed with 1% acetic acid (novel context).

For foot shock application (day 0), mice were placed into the conditioning chamber for 3 min. After 180 s, a sine wave tone (80 dB, 9 kHz) was presented for 20 s, which co-terminated with a 2 s scrambled electric foot shock of 1.5 mA. The mice remained in the shock chamber for another 60 s. To measure the freezing responses to the tone, mice were placed into the novel environment (cylinder) on the following day (day 1). Three minutes later, a 3 min tone was presented (80 dB, 9 kHz). The animals were returned to their home cages 60 s after the end of tone presentation. Contextual fear was tested by re-exposing the animals to the shock context for 3 min on day 2. In order to assess potential differences in long-term memory, all animals were exposed to the novel and familiar (shock) context 30 days later (days 31 and 32 respectively). As a measure of fear memory, freezing behavior was recorded and analyzed by an observer blind to genotype. Freezing was scored if the animals adopted an immobile posture (except for breathing-related movement) with all four paws on the ground and the head in a horizontal position. Data were analyzed in 60 s bins and normalized to the observation interval.

### Chronic Social Defeat Stress Paradigm

The chronic social defeat stress (CSDS) paradigm is commonly utilized to induce anxiety- and depression-related endophenotypes in mice and was performed as previously described (Wagner et al., 2011; Wang et al., 2011; Hartmann et al., 2012a, 2016; Gassen et al., 2014; Metzger et al., 2017; Dedic et al., 2018b). *Crh*^*Ctrl*^ and *Crh^*CKO–GABA*^* mice (10–15 male mice per group between 3 and 4 months of age) were submitted to CSDS for 21 consecutive days. They were introduced into the home cage (45 cm × 25 cm) of a dominant CD1 resident for no longer than 5 min during which they experienced multiple bouts of defeat. Following defeat, animals spent 24 h in the same cage, which was separated via a perforated steel partition, enabling sensory but not physical contact. Every day experimental mice were exposed to a new unfamiliar resident. Defeat encounters were randomized, with variations in starting time in order to decrease the predictability to the stressor and minimize habituation effects. Control animals were housed in their home cages throughout the course of the experiment. Control and stressed mice were housed in the same room, but in different racks. All animals were handled daily and weighed. Behavioral testing was conducted during the last week of the CSDS paradigm in the following order: OF test, Social avoidance test, EPM, DaLi, and the FST. For evaluation of the corticosterone response to an acute stressor, blood samples were collected 30 min (response levels) after the start of FST by tail cut. All animals were killed by an overdose of isoflurane at the end of the experiment (1 day after the last CSDS encounter). Trunk blood was collected after decapitation for the assessment of basal corticosterone levels. Adrenal and thymus glands were removed, dissected from fat, and weighed.

### Assessment of Immediate Early Gene (IEG) Expression

Stress-induced expression of IEGs c-fos and zif268 (also known as Krox-24, NGF1-A, Egr1, TIS8, and Zenk), was assessed in naïve *Crh*^*Ctrl*^ and *Crh*^*CKO–GABA*^ mice following FST. The animals were euthanized 30 min following the onset of the FST. The brains were carefully removed and immediately shock-frozen on dry ice and stored at −80°C until further processing for ISH.

### Statistical Analyses

Statistical analyses were performed using the commercially available software SPSS v16.0 (SPSS, Chicago, IL, United States) and GraphPad Prism v7.0 (GraphPad Software, La Jolla, CA, United States). All results are presented as mean ± s.e.m. Statistical significance was defined as *p* < 0.05. Normality and equality of variance were analyzed with the D’Agostino-Pearson omnibus test and Bartlett’s test, respectively. In cases where sample sizes were too small, data distribution was assumed to be normal. All data were tested for outliers using the Grubbs’ test. Based on the results of these tests, appropriate parametric (two-tailed unpaired *t*-test) or non-parametric (Mann–Whitney *U*-test) tests were performed. For CSDS experiments, the effects of genotype and condition on all other behavioral and neuroendocrine parameters were assessed by two factorial ANOVA (2-way ANOVA). Time-dependent measures were analyzed with repeated-measures ANOVA followed by Bonferroni *post hoc* analysis. Whenever significant main or interaction effects were found by the ANOVAs, Bonferroni *post hoc* tests were carried out to locate simple effects. Conditional knockout mice and control littermates were assigned to the experimental group on the basis of genotype. Age-matched littermates were used as controls in all experiments. Animals were allocated to the experimental groups in a semi-randomized manner and data analysis was performed blinded to the group allocation.

## Results

### *Crh* mRNA Expression Mapping in *Crh*^*CKO–GABA*^ Mice

**FIGURE 1:**
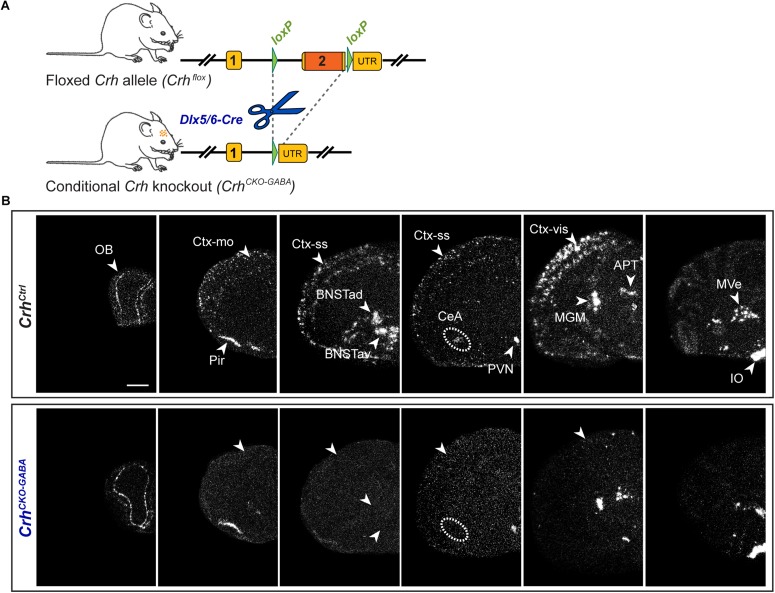
*Crh* deletion pattern in conditional *Crh*^*CKO–GABA*^ mice. **(A)** Schematic representation of the previously established conditional inactivation of the *Crh* gene (Dedic et al., 2018a). Exon 2 is flanked by *loxP* sites. Breeding of *Crh*^*flox*^ mice to *Dlx5/6-Cre* driver mice resulted in *Crh* deletion specifically in GABAergic forebrain neurons. UTR, untranslated region. **(B)** Expression of *Crh* mRNA was assessed by *ISH* in control and *Crh*^*CKO–GABA*^ mice using a riboprobe, which detects the 3′UTR of *Crh*. Dark-field photomicrographs depict the specific *Crh* deletion pattern in *Crh*^*CKO–GABA*^ mice. Areas of interest are highlighted with arrowheads and dashed lines. Images are representative of three separate experiments. APT, anterior pretectal nucleus; CeA, central amygdala; BNSTad/v, anterior bed nucleus of the stria terminalis dorsal/ventral; CtxII/III, CtxV/VI, cortical layers; IO, inferior olive; MGM, medial geniculate nucleus; MVe, medial vestibular nucleus; Ctx-mo, motor cortex; OB, olfactory bulb; Pir, piriform cortex; PVN, paraventricular nucleus of the hypothalamus; Ctx-ss, somatosensory cortex; Ctx-vis, visual cortex. Scale bar represents 1 mm.

Using sensitive *in situ* hybridization (ISH) methods, we recently mapped the neurochemical identity of *Crh* neurons across the mouse brain, revealing an overwhelming majority of GABAergic (*Gad65/67*-positive) CRH neurons in the cortex, hippocampus, central nucleus of the amygdala (CeA) and the bed nucleus of the stria terminalis (BNST), which has also been reported by others (Kubota et al., 2011; Kubota, 2013; Dedic et al., 2018a; Gunn et al., 2019). In contrast, *Crh* neurons in the Pir and PVN primarily coexpressed the glutamatergic markers *Vglut1* and *Vglut2*, respectively (Dedic et al., 2018a). In order to dissect the role of *Crh* in GABAergic circuits we crossed the recently generated *Crh*^*flox/flox*^ mice with *Dlx5/6-Cre* transgenic mice (Rodríguez et al., 2000; Kamprath et al., 2006; Monory et al., 2006), in which *Cre* is driven by the regulatory sequences of the *Dlx5/Dlx6* homeobox genes expressed in migrating, forebrain GABAergic neurons during development (E10). Consequently, the resulting *Crh* deletion pattern in *Crh^*CKO–GABA*^* mice was restricted specifically to GABAergic neurons of the forebrain (Figure 1) and was largely in line with the previously published *Crh* expression maps obtained with double ISH (Dedic et al., 2018a). Thus, absence of *Crh* mRNA expression was observed in the CeA, hippocampus and throughout the cortex of *Crh^*CKO*–GABA^* mice (Figure 1B). Expectedly, *Crh* mRNA levels were comparable between control and *Crh*^*CKO–GABA*^ mice in the Pir, PVN and throughout the hindbrain and brainstem areas.

### HPA Axis Activity, Baseline Locomotion, Anxiety, and Fear Memory Are Not Altered in *Crh^*CKO*–*GABA*^* Mice

**FIGURE 2:**
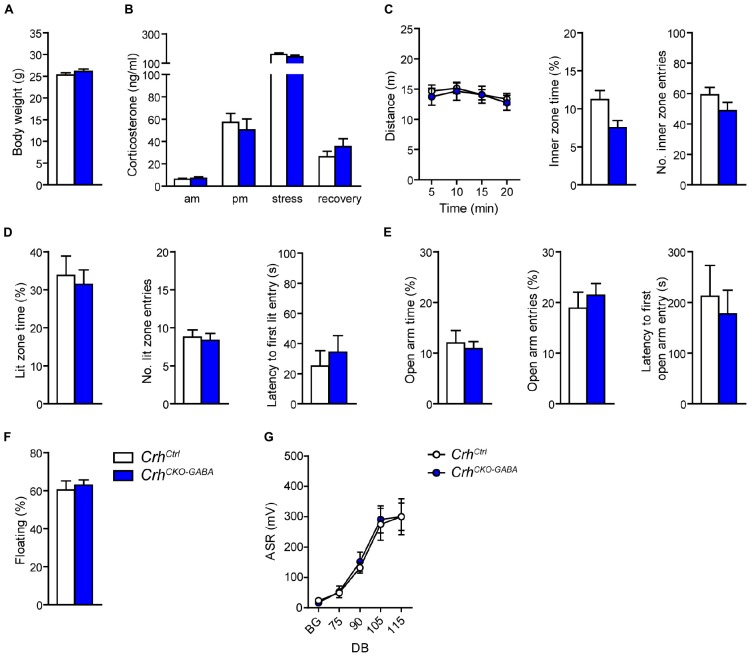
*Crh* deletion in GABAergic forebrain neurons does not alter HPA axis activity, locomotion and anxiety-related behavior under baseline conditions. Body weight **(A)** as well as circadian and stress-induced corticosterone release **(B)** were not significantly changed in *Crh*^*CKO–GABA*^ mice compared to littermate controls. Similarly, spontaneous locomotion in the OF test **(C)**, anxiety-related behavior in the DaLi **(D)** and EPM **(E)**, immobility in the FST **(F),** and the acoustic startle response **(G)** did not significantly differ between genotypes. Unpaired two-tailed *t*-test; time- and decibel-dependent measures: repeated-measures ANOVA; *p* < 0.05; *n* = 12–15/group). Data are shown as mean ± s.e.m.

In order to assess whether lack of *Crh* from GABAergic forebrain neurons would affect HPA axis activity, we measured corticosterone release in *Crh^*CKO*–GABA^* mice (Figure 2B). No differences in plasma corticosterone levels were detected between *Crh^*CKO*–GABA^* mice and control littermates in the morning (am: unpaired *t*-test, *t*_26_ = 0.57, *p* = 0.6), evening (pm: unpaired *t*-test, *t*_22_ = 0.19, *p* = 0.85), as well as 10 and 90 min after restraint stress (stress: unpaired *t*-test, *t*_24_ = 1.1, *p* = 0.29/recovery: unpaired *t*-test, *t*_19_ = 1.15, *p* = 0.27). In addition, no significant, genotype-mediated changes were observed in body weight (unpaired *t*-test, *t*_27_ = 1.1, *p* = 0.3; Figure 2A). To functionally dissect whether GABAergic neurons are mediating the effects of CRH on aspects of emotional behavior, *Crh^*CKO*–GABA^* mice were subjected to a series of behavioral tests. In the Open field (OF) test, locomotor activity [repeated measures ANOVA, genotype *F*_(1, 27)_ = 0.12, *p* = 0.73], inner zone time (unpaired *t*-test, *t*_27_ = 1.7, *p* = 0.1) and the number of inner zone entries (unpaired *t*-test, *t*_27_ = 1.0, *p* = 0.3) were not significantly altered in *Crh^*CKO*–GABA^* mice compared to controls (Figure 2C). Interestingly, *Crh^*CKO*–GABA^* mice displayed no changes in anxiety-related behavior (Figures 2D,E) assessed in the dark/light box (DaLi) and elevated plus maze (EPM) test [DaLi, unpaired *t*-test: lit zone time (%), *t*_25_ = 0.42, *p* = 0.6; lit zone entries, *t*_25_ = 0.46, *p* = 0.6; latency lit zone time (%), *t*_25_ = 0.61, *p* = 0.5/EPM, unpaired *t*-test: open arm time (%), *t*_22_ = 0.4, *p* = 0.7; open arm entries (%), *t*_22_ = 0.66, *p* = 0.5; latency, *t*_22_ = 0.46, *p* = 0.7]. Similarly, immobility in the FST did not differ between *Crh^*CKO*–GABA^* mice and littermate controls (unpaired *t*-test, *t*_27_ = 0.46, *p* = 0.65; Figure 2F).

**FIGURE 3:**
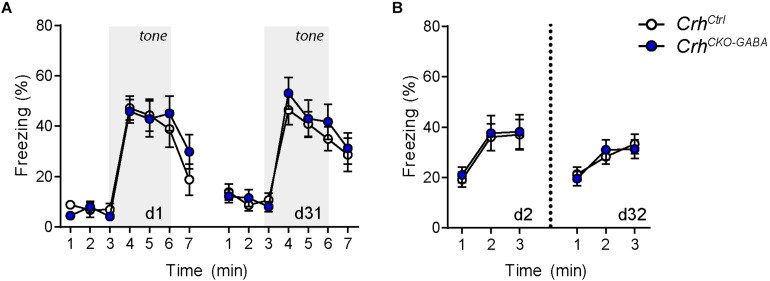
Cued and context-dependent fear condition is not altered in *Crh*^*CKO–GABA*^ mice. **(A)** Auditory fear memory assessed one and 30 days after conditioning was not significantly altered in *Crh*^*CKO–GABA*^ mice compared to littermate controls. Both groups expressed similar levels of freezing in the novel context under baseline conditions (initial 3 min) and following exposure to the tone (3–6 min). **(B)** Expression of contextual fear memory was also not altered between genotypes. Repeated-measures ANOVA; *p* < 0.05; *n* = 13/group). Data are shown as mean ± s.e.m.

The CRH system has been implicated in modulating aspects of the acoustic startle response, although both increased and decreased startle amplitudes have been observed following CRH overexpression and/or injection (Lee and Davis, 1997; Groenink et al., 2002, 2008; Flandreau et al., 2015). Control and *Crh^*CKO*–GABA^* mice exhibited a characteristic, decibel-dependent increase in the acoustic startle response (Figure 2G), but no differences were observed between genotypes [repeated measures ANOVA; db *F*_(1, 6)_ = 39.1, *p* < 0.0001; genotype *F*_(1, 24)_ = 0.04, *p* = 0.8]. In view of CRH’s important role in conditioned fear (Haubensak et al., 2010; Isogawa et al., 2013; Tovote et al., 2015; Sanford et al., 2016; Fadok et al., 2017; Dedic et al., 2018a), we additionally assessed auditory and contextual fear memory. *Crh^*CKO*–GABA^* mice showed no significant changes in tone- and context-dependent freezing compared to control littermates (Figure 3). This was also the case when re-tested 30 days later, suggesting that long term fear memory is also not altered in *Crh^*CKO*–GABA^* mice [repeated measures ANOVA; tone: genotype *F*_(1, 24)_ = 0.06, *p* = 0.8; tone d30: genotype *F*_(1, 23)_ = 0.19, *p* = 0.7/context: genotype *F*_(1, 24)_ = 0.05, *p* = 0.8; context d30: genotype *F*_(1, 23)_ = 0.006, *p* = 0.9]. Overall, deletion of *Crh* from GABAergic forebrain neurons did not produce significant differences in HPA axis activity or any of the evaluated behavioral endpoints.

### *Crh*^*CKO–GABA*^ Mice Exhibit Deficits in Baseline Social Behavior and Resilience to Chronic Social Defeat Stress

Recent work has suggested that the CRH/CRHR1 system might exert different effects on emotional valance under physiological and severe stress conditions (Lemos et al., 2012; Dedic et al., 2018a). To assess whether deletion of *Crh* from GABAergic forebrain neurons would alter stress susceptibility, *Crh^*CKO*–GABA^* mice were subjected to 3 weeks of CSDS, a paradigm commonly applied to induce anxiety- and depression-related endophenotypes in mice (Berton et al., 2006; Golden et al., 2011; Gassen et al., 2014; Hartmann et al., 2016; Dedic et al., 2018b).

**FIGURE 4:**
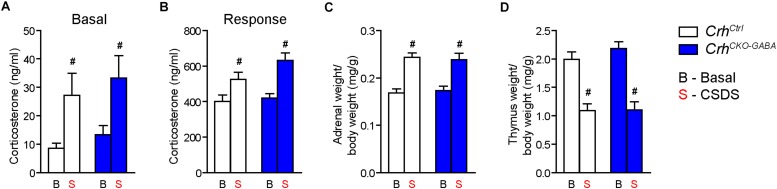
CSDS induced similar neuroendocrine and physiological alterations in *Crh*^*CKO–GABA*^ and control mice. **(A)** Control and *Crh*^*CKO–GABA*^ mice showed increased basal corticosterone levels following exposure to 3 weeks of CSDS. **(B)** Corticosterone response levels assessed 30 min after a FST challenge, were significantly increased in chronically stressed mice independent of genotype. The efficacy of the CSDS paradigm was further demonstrated by a significant enlargement of the adrenal glands **(C)** and a substantial decrease in thymus size **(D)** in chronically stressed mice. Two-way ANOVA + Bonferroni *post hoc* test; ^#^significantly different from the basal condition of the same genotype, *p* < 0.05; *n* = 10–15/group. Data are shown as mean ± s.e.m.

**FIGURE 5:**
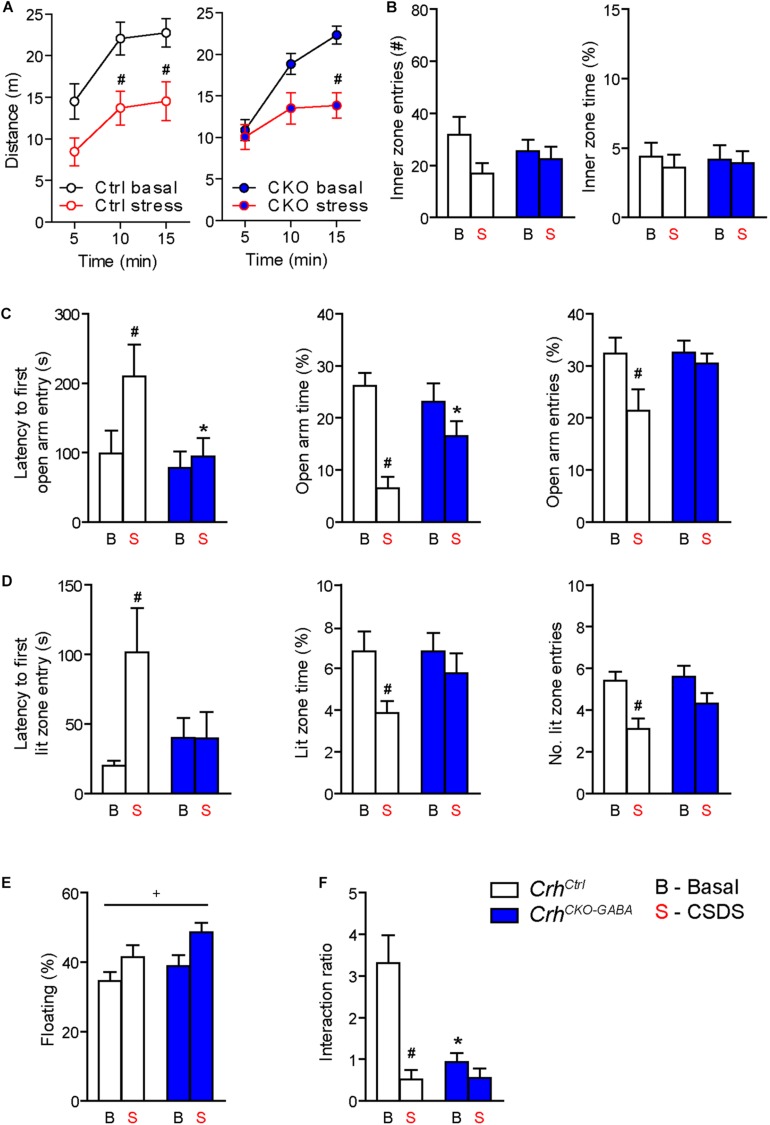
*Crh* deletion from forebrain GABAergic neurons induces social deficits under baseline conditions and reduces the susceptibility to CSDS-induced anxiety. **(A,B)** CSDS reduced locomotion in the OF test, independent of genotype. Inner zone time and the number of inner zone entries were not significantly affected by stress or genotype. Following CSDS, anxiety-related behavior in the DaLi **(C)** and EPM **(D)** was significantly increased in control but not *Crh*^*CKO–GABA*^ mice. This is depicted by increased latencies to enter the aversive lit zone of the DaLi and open arms of the EPM, as well as significantly reduced lit/open arm times and entries in stressed control but not stressed *Crh*^*CKO–GABA*^ mice. **(E)** CSDS increased immobility in the FST independent of genotype. **(F)** CSDS reduced interaction ratios in control mice during the social avoidance test. This effect was absent in *Crh*^*CKO–GABA*^ mice, which displayed a significant reduction in social interaction already under baseline conditions. Two-way ANOVA or repeated measures ANOVA + Bonferroni *post hoc* test; ^∗^ Significant from control of the same condition, ^#^significant from the basal group of the same genotype, ^+^significant condition effect; *p* < 0.05; *n* = 10–15/group. Data are shown as mean ± s.e.m.

Basal (Figure 4A) and acute-stress induced corticosterone levels (Figure 4B) were significantly elevated in chronically stressed mice independent of genotype [Basal: 2-way ANOVA, stress *F*_(1, 46)_ = 10.4, *p* < 0.005; Bonferroni *post hoc* test, *p* < 0.05/Response: 2-way ANOVA, stress *F*_(1, 44)_ = 20.4, *p* < 0.0001; Bonferroni *post hoc* test, *p* < 0.05]. In addition, CSDS resulted in enhanced adrenal gland weight and decreased thymus weight in stressed *Crh*^*Ctrl*^ and *Crh*^*CKO–GABA*^ mice [AG: 2-way ANOVA, stress *F*_(1, 48)_ = 42.5, *p* < 0.0001; Bonferroni *post hoc* test, *p* < 0.05/Thymus: 2-way ANOVA, stress *F*_(1, 48)_ = 58.9, *p* < 0.0001; Bonferroni *post hoc* test, *p* < 0.05; Figures 4C,D). These robust physiological and neuroendocrine changes evoked by CSDS validate the efficacy of the paradigm and are in line with previous studies (Wagner et al., 2011; Hartmann et al., 2012a, b, 2016; Gassen et al., 2014; Metzger et al., 2017; Dedic et al., 2018b). Spontaneous locomotion in the OF tests was significantly reduced in *Crh*^*Ctrl*^ and *Crh*^*CKO–GABA*^ mice following CSDS (Figure 5A), whereas inner zone time and number of entries were not affected [repeated measures-ANOVA, time × stress *F*_(1, 47)_ = 4.8, *p* < 0.05; stress *F*_(1, 48)_ = 16.9, *p* < 0.0001; Bonferroni *post hoc* test, *p* < 0.05; Figure 5B]. Locomotion was not significantly altered by genotype, both under basal and chronic stress conditions. The OF test was conducted under low illumination (15 lux) in order to minimize potential effects of anxiety on locomotion. This likely explains the lack of CSDS effects on inner zone time and number of entries.

Interestingly, *Crh*^*CKO–GABA*^ mice were less susceptible to the anxiety-inducing effects of CSDS (Figures 5C,D). The latencies to enter the aversive lit zone of the DaLi and open arms of the EPM were significantly increased in chronically stressed *Crh*^*Ctrl*^ but not *Crh*^*CKO–GABA*^ mice [DaLi: 2-way ANOVA, genotype × stress *F*_(1, 43)_ = 4.3, *p* < 0.05; stress *F*_(1, 43)_ = 4.2, *p* < 0.05; Bonferroni *post hoc* test, *p* < 0.05/EPM: 2-way ANOVA, genotype *F*_(1, 44)_ = 4.6, *p* < 0.05; stress *F*_(1, 44)_ = 4.0, *p* = 0.051; Bonferroni *post hoc* test, *p* < 0.05]. In accordance, lit zone time and number of entries were significantly reduced in controls following CSDS; this stress effect was absent in *Crh*^*CKO–GABA*^ mice [time: 2-way ANOVA, stress *F*_(1, 44)_ = 4.9, *p* < 0.05; Bonferroni *post hoc* test, *p* < 0.05/entries: 2-way ANOVA, stress *F*_(1, 44)_ = 12.2, *p* < 0.005; Bonferroni *post hoc* test, *p* < 0.05]. These results indicate that CRH in GABAergic neurons modulates the effects of CSDS on anxiety-related behavior. In addition, immobility in the FST was increased in chronically stressed mice, independent of genotype [2-way ANOVA, stress *F*_(1, 48)_ = 12.2, *p* < 0.05; Figure 5E). As initially observed, no genotype effects were detected under baseline conditions in the DaLi, EPM, and FST.

Alterations in social behavior are observed in many psychiatric disorders including major depressive disorder, bipolar disorder, schizophrenia and autism (Nestler and Hyman, 2010; Kaiser and Feng, 2015). In addition, CSDS has repeatedly been shown to reduce social interaction and enhance avoidance behavior in rodents (Berton et al., 2006; Golden et al., 2011; Dedic et al., 2018b). To test whether *Crh* deletion from GABAergic forebrain neurons would affect social behavior under basal and chronic stress conditions, we performed the social avoidance test. Chronically stressed control mice spent significantly less time in close proximity to a social counterpart, indicated by a decreased interaction ratio (Figure 5F). Interestingly, compared to controls, *Crh*^*CKO–GABA*^ mice exhibited reduced social interaction already under basal conditions, which was not further aggravated following CSDS [2-way ANOVA, genotype × stress *F*_(1, 41)_ = 9.8, *p* < 0.005; stress *F*_(1, 41)_ = 17.0, *p* < 0.0005; genotype *F*_(1, 41)_ = 9.3, *p* < 0.005; Bonferroni post-test, *p* < 0.05]. This suggests that CRH in GABAergic neurons is required for the expression of “normal” social behavior.

### Stress-Induced Expression of Immediate Early Genes Is Reduced in *Crh*^*CKO–GABA*^ Mice

**FIGURE 6:**
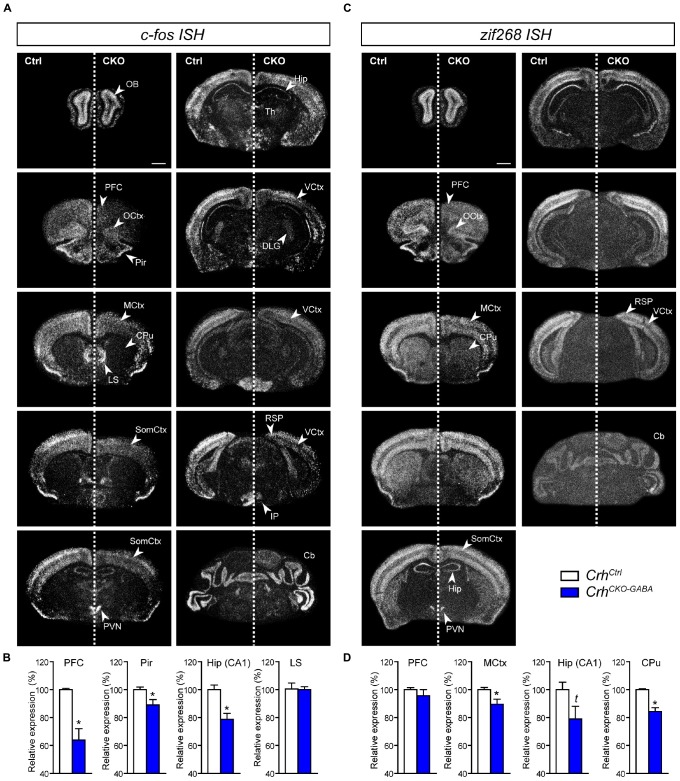
Stress-induced neuronal activation is reduced in *Crh*^*CKO–GABA*^ mice. Brain-wide *c-fos* **(A)** and *zif268* **(C)** mRNA expression pattern in control (Ctrl) and conditional knockout (CKO) mice determined with *ISH* 30 min after acute stress (6 min FST). Quantification of the respective *c-fos* **(B)** and *zif268* **(D)** mRNA expression in distinct brain regions is shown below the representative dark-field photomicrographs. Cb, cerebellum; CPu, caudate putamen; DLG, dorsolateral geniculate nucleus; Hip, CA1 of dorsal hippocampus; LS, lateral septum; MCtx, motor cortex; OB, olfactory bulb; OCtx, orbital cortex; PVN, paraventricular nucleus of the hypothalamus; Pir, piriform cortex; PFC, prefrontal cortex; RSP, retrosplenial area; SomCtx, somatosensory cortex; TH, thalamus; VCtx, visual cortex. ^∗^Significantly different from control; unpaired, two-tailed *t*-test, *p* < 0.05; t trend, *p* ≤ 0.1; *n* = 5–6/group. Data are shown as mean ± s.e.m. Scale bar represents 1 mm.

Following the observation that lack of *Crh* from forebrain GABAergic neurons promotes resilience to CSDS, we asked whether stress would induce similar patterns of neuronal activation in control and *Crh*^*CKO–GABA*^ mice. Thus, the mRNA expression of the immediate early genes *c-fos* and *zif268* was analyzed in naïve animals 30 min after exposure to an acute stressor (6 min of FST) using ISH. *C-fos* and *zif268* have been commonly used to map neuronal activity in different brain regions of various species. Both are rapidly and transiently induced by a variety of stimuli including stress, and show overlapping as well as distinct expression patterns following specific stimuli (Sagar et al., 1988; Sheng and Greenberg, 1990; Cullinan et al., 1995; Guzowski et al., 2001; Leslie and Nedivi, 2011; Veyrac et al., 2014; Farina and Commins, 2016; Gallo et al., 2018; Bisagno and Cadet, 2019). *C-fos* and *zif268* expression is consistently low under baseline conditions and was not significantly different between control and *Crh*^*CKO–GABA*^ mice (data not shown). Following acute FST-stress, a marked increase of *c-fos* and *zif268* expression was detected throughout the brain of *Crh*^*Ctrl*^ and *Crh*^*CKO–GABA*^ mice (Figure 6). However, diminished *c-fos* expression was observed in the dorsal hippocampus and most cortical areas of *Crh*^*CKO–GABA*^ compared to control animals (Figures 6A,B). Similar effects were observed for *zif268*, where decreased activation was additionally detected in the caudate putamen of *Crh*^*CKO–GABA*^ mice compared to controls (Figures 6C,D). These results demonstrate that CRH depletion from GABAergic neurons attenuates stress-induced neuronal activity changes in CRHR1-expressing brain regions such as the cortex and hippocampus. This is further supported by the fact that *c-fos* expression was not differentially altered in the PVN of *Crh*^*CKO–GABA*^ mice, a structure largely devoid of CRHR1 and CRHR2 (Van Pett et al., 2000). Our findings suggest that deletion of *Crh* in GABAergic neurons protects from the adverse effects of CSDS, possibly by reducing stress-induced neuronal activation.

## Discussion

The central peptidergic CRH/CRHR1 system impinges on a broad spectrum of physiological processes that are the basis for successful adaptation and concomitantly integrate autonomic, neuroendocrine, and behavioral stress responses. Accordingly, dysregulation of the CRH/CRHR1 system has been observed in stress-related psychopathologies including depression and anxiety disorders (Laryea et al., 2012; Deussing, 2013; Janssen and Kozicz, 2013; Henckens et al., 2016; Dedic et al., 2017). It has widely been accepted that CRH-CRHR1 signaling promotes the stress response and aversive behaviors, but more recent findings provide clear evidence for a requirement of specific CRH circuits in positive emotional valance and appetitive responses (Lemos et al., 2012; Dedic et al., 2018a). Thus, a more complex picture has emerged, suggesting that there are brain region- and cell type-specific effects of CRH/CRHR1 signaling that are influenced by the individual’s prior experience and that shape molecular, cellular and ultimately behavioral responses to stressful challenges (Henckens et al., 2016; Dedic et al., 2017).

We recently demonstrated that a specific subpopulation of VTA-projecting, GABAergic CRH neurons in the extended amygdala (CeA and BNST) act anxiety-suppressing by positively modulating dopamine release under physiological conditions (Dedic et al., 2018a). These neurons are characterized by the co-expression of *Camk2*α and account for approximately one third of the CRH population in the CeA and BNST. Since absence of *Crh* specifically in GABAergic, *Camk2a*-expressing CRH neurons increased anxiety, we wondered whether deletion of the neuropeptide from the entire GABAergic forebrain population would produce a similar or distinct phenotype. Moreover, we addressed whether *Crh*^*CKO–GABA*^ mice would display differential behaviors under baseline and chronic stress conditions. Mapping analyses in *Crh*^*CKO–GABA*^ mice showed complete lack of *Crh* mRNA in the CeA, BNST, hippocampus and throughout most of the cortex, which is in line with previous studies reporting predominant expression of cortical and limbic CRH in GABAergic neurons (Chen et al., 1998, 2001; Kubota et al., 2011; Kubota, 2013; Dedic et al., 2018a; Gunn et al., 2019). To our initial surprise, *Crh*^*CKO–GABA*^ mice showed no gross behavioral changes in locomotion, anxiety, startle response and fear memory, but exhibited social deficits under baseline conditions. This is partially in line with earlier studies reporting normal baseline locomotor activity, exploration, anxiety, startle response and learning in conventional *Crh* knockout (*Crh* null) mice (Weninger et al., 1999). In contrast, specific deletion of *Crh* from GABAergic, *Camk2*α-expressing neurons increases anxiety and fear memory expression (Dedic et al., 2018a). A possible explanation for the discrepancy in anxiety- and fear-related behavior between the two mouse lines could be the fact that *Crh*^*CKO–GABA*^ mice lack *Crh* in GABAergic circuits that modulate positive and negative emotional valance. In addition, *Camk2*α*-CreERT2*-induced deletion of *Crh* is initiated during adulthood as opposed to *Dlx5/6-Cre*-driven recombination in *Crh*^*CKO–GABA*^ mice, which occurs during embryonic development (E10). Thus, differences in deletion time points and/or subsequent compensatory mechanisms between *Crh^*CKO–Camk*2α^* and *Crh*^*CKO–GABA*^ mice might account for the difference in anxiety-related behavior. Along these lines, mice carrying a PVN-restricted deletion of *Crh* (*Sim1CrhKO*) exhibit reduced anxiety under baseline conditions (Zhang et al., 2017). These suggest that PVN CRH neurons are likely modulating aversive responses in naïve animals, which has also been observed by others (Füzesi et al., 2016; Kim et al., 2019). The lack of effect on baseline anxiety in *Crh*^*CKO–GABA*^ mice further support this, given the fact *Crh* expression is absent from cortical and limbic regions but preserved in the PVN of these animals.

Overexpression of CRH has repeatedly been shown to induce stress-like behavioral responses including increased fear and anxiety under baseline conditions (Stenzel-Poore et al., 1994; Van Gaalen et al., 2002; Lu et al., 2008; Kolber et al., 2010; Dedic et al., 2012; Flandreau et al., 2012). However, overexpression studies are limited in their ability to accurately reflect the endogenous function of specific CRH subpopulations, which is primarily due to ectopic and non-physiological expression of the peptide in the brain. In addition, corticosterone release is dysregulated in many CRH overexpressing lines as well as CRH null mice (Muglia et al., 1995; Dedic et al., 2018a), which can obscure the interpretations of the behavioral results. In contrast, HPA axis activity was not significantly altered in *Crh^*CKO–GABA*^* mice, due to the lack of *Dlx5/6-Cre* mediated recombination in CRH-expressing neurons of the PVN. This suggests that CRH drive in the PVN, rather than other extrahypothalamic sites, is primarily responsible for regulating HPA axis function.

Interestingly, *Crh*^*CKO–GABA*^ mice exhibited significantly reduced social interaction (increased social avoidance) compared to controls under basal conditions. This implies a requirement of CRH in forebrain GABAergic neurons for the expression of “normal” social behavior. These observations are partially in line with the work of Kasahara and colleagues, which demonstrated enhanced social investigation in CRH overexpressing mice (Kasahara et al., 2011). In contrast, BNST and amygdala-specific CRH administration were shown to decrease social interaction (Dunn and File, 1987; Sajdyk et al., 1999; Lee et al., 2008). Along these lines, CRH infusion into the nucleus accumbens propagates stress-induced social avoidance (mimicked by optogenetic activation of VTA-NAc projections). However, in the absence of stress or optical stimulation, CRH application to the NAc produced no alterations in social behavior (Walsh et al., 2014). Similarly, knockdown of *Crh* in the PVN attenuated social avoidance in chronic social defeated mice, without altering social interaction under basal conditions (Elliott et al., 2010). However, possible alterations in HPA axis function, which could have been caused by *Crh* knockdown in the PVN, were not assessed in that study. Thus, it cannot be excluded that the observed phenotype is partially caused by reduced glucocorticoid levels. Importantly, results obtained after exogenous CRH application have to be interpreted with caution considering that these experiments are only mimicking acute effects of CRH hyperdrive. In addition, enhanced receptor activation following exogenous CRH application or overexpression might overshadow normal patterns of endogenous CRH release. Furthermore, co-activation of CRHR2 might obscure the relevance of CRH-CRHR1 signaling in social behavior. This is supported by the fact that *Ucn3*-, *Ucn2-* (specific CRHR2 ligands), and *Crhr2*-deficient mice display alterations in social behavior (Deussing et al., 2010; Breu et al., 2012). Importantly, social avoidance/approach paradigms are often confounded by anxiety, such that anxious animals are more likely to display reduced social engagement. This was not the case for *Crh*^*CKO–GABA*^ mice, which displayed no alterations in anxiety under baseline conditions.

In accordance with recent studies, our data further support the presence of distinct CRH circuits that have the ability to positively regulate emotional valance under physiological (non-stress) conditions. Importantly, severe stress was previously reported to switch the action of CRH from appetitive to aversive (Lemos et al., 2012). Specifically, CRH produces an appetitive effect in the nucleus accumbens under basal conditions, which resulted from CRH’s ability to positively regulate dopamine release (Lemos et al., 2012). However, repeated stress exposure was shown to induce a persistent dysregulation of CRH-dopamine interactions that normally produce a positive affective state, resulting in an aversive phenotype. Along these lines, we showed that enhanced CRH/CRHR1 signaling in the VTA produces an anxiolytic effect in naïve animals (Dedic et al., 2018a); an effect that is lost following chronic drug exposure, in which CRH action in the VTA becomes aversive/anxiogenic (George et al., 2012; Grieder et al., 2014). In addition to the effects on emotional and reward behavior, recent experiments showed that CRH within the inferior olive of the brain stem modulates motor capabilities of mice under challenging, but not baseline conditions (Ezra-Nevo et al., 2018). In line of these results, we investigated whether deletion of *Crh* from forebrain GABAergic neurons might confer resilience to a severe, long-term stressor. Following exposure to CSDS, increased anxiety-related behavior was detected in control but not *Crh*^*CKO–GABA*^ mice. These findings are in line with a large body of work demonstrating the ability of CRHR1 antagonists to block stress-induced behavioral alterations (Heinrichs et al., 1992; Swiergiel et al., 1993; Liebsch et al., 1995; Shaham et al., 1998; Weninger et al., 1999; Habib et al., 2000; Gully et al., 2002; Zorrilla et al., 2002; Ducottet et al., 2003; Robison et al., 2004; Roozendaal et al., 2008; Bruijnzeel et al., 2009; Ivy et al., 2010; Gilpin et al., 2016). Along these lines, forebrain-specific deletion of *Crhr1* decreases the susceptibility to chronic stress-induced cognitive deficits (Wang et al., 2011, 2012, 2013). Of note, the protective effects in *Crh*^*CKO–GABA*^ mice were specifically observed for anxiety behavior. Neither locomotion, immobility in the FST or any of the physiological parameters were differentially affected by CSDS in control and *Crh*^*CKO–GABA*^ mice. Thus, the effects on anxiety were independent of altered locomotion or HPA axis function. Social avoidance in *Crh*^*CKO–GABA*^ mice was not further aggravated by CSDS most likely due to the established floor effect under basal conditions.

Subsequent molecular assessment in *Crh*^*CKO–GABA*^ mice revealed a significant reduction in stress-induced mRNA levels of immediate early genes *c-fos* and *zif268* in CRHR1-expressing brain regions, including the hippocampus and cortex. Whether lack of *Crh* from GABAeric forebrain neurons induces compensatory regulation of CRHR1, CRHR2, and Urocortin expression remains to be investigated. Overall, the results in *Crh*^*CKO–GABA*^ mice mirror the activating effects of central CRH application on *c-fos* expression (Dunn and Berridge, 1990; Liebsch et al., 1995; Benoit et al., 2000; Bittencourt and Sawchenko, 2000; Rostkowski et al., 2013; Wiersielis et al., 2016; Salvatore et al., 2018). Along these lines, CRHR1-antagonist treatment block CRH- and stress-induced increases in c-fos expression (Doyon et al., 2007; Skórzewska et al., 2008). Given its ability to facilitate excitatory neurotransmission in regions such as the amygdala and hippocampus, CRH is generally denoted as an activating neuropeptide (Aldenhoff et al., 1983; Hollrigel et al., 1998; Giesbrecht et al., 2010; Refojo et al., 2011; Kratzer et al., 2013). As previously mentioned, CRH administration into different brain regions can induce c-fos expression and mimic acute-stress effects. In addition, CNS-specific CRH overexpressing mice demonstrate enhanced *c-fos* and *zif268* activation of the locus coeruleus following forced-swim stress (Lu et al., 2008). Thus, it seems plausible that deletion of *Crh* from most cortical and all limbic regions would produce a net inhibitory effect, exhibited by decreased stress-induced *c-fos* activation. Dampened neuronal activity in response to an acute challenge likely underlies aspects of stress-resilience in *Crh*^*CKO–GABA*^ mice. The necessity to uncover the precise mechanisms by which CRH acts in an excitatory fashion becomes evident inline of the fact that CRH is primarily released from inhibitory GABAergic neurons.

It is still not entirely understood how sensory information is represented and evaluated by specific CRH/CRHR1 circuits to produce discrete autonomic and behavioral outputs. An earlier study reported demethylation of the CRH-promoter region, which resulted in enhanced *Crh* gene expression in the PVN of mice that were susceptible to chronic defeat stress (Elliott et al., 2010). Knockdown of *Crh* in the PVN attenuated defeat-induced behavioral alterations. To what extent CSDS alters *Crh* methylation in other brain regions remains to be explored. Thus, epigenetic regulation of CRH likely constitutes a mechanism by which the brain regulates long-term behavioral responses to stress. Moreover, stress was shown to induce metaplasticity in PVN CRH neurons at glutamate synapses, which ultimately primed behavioral and physiological responses (Sterley et al., 2018). In addition, recent calcium imaging experiments revealed the ability of PVN CRH neurons to respond in a rapid and biphasic manner to encode positive and negative valance of specific stimuli (Kim et al., 2019). More specifically, PVN CRH neurons are immediately activated by aversive cues (e.g., FST, predator odor or food deprivation) and are rapidly suppressed by appetitive stimuli (e.g., palatable food or social interaction). These findings are consistent with previous work showing that activation of hypothalamic and amygdalar CRH circuits results in aversive or fear-related responses (Füzesi et al., 2016; Sanford et al., 2016; Fadok et al., 2017; Zhang et al., 2017; Sterley et al., 2018). However, it will be important to address whether the diverse behavioral effects are actually driven by changes in CRH release or potentially modulated by other, co-expressed neurotransmitters and/or neuromodulators.

In addition to the repeatedly described limbic CRH networks that drive aversive behavioral responses, our current and previous work have revealed the presence of specific GABAergic CRH circuits that modulate positive emotional and social valance under baseline conditions. This suggests the presence of distinct GABAergic CRH circuits that function in a concerted but antagonistic manner to keep emotional responses under physiological conditions in balance. This concept was initially observed for CRHR1, showing that CRHR1-controlled glutamatergic and dopaminergic circuits modulate anxiogenic and anxiolytic responses under baseline conditions (Refojo et al., 2011). Whether CRHR1 signaling in dopaminergic neurons retains anxiety-suppressing properties following exposure to chronic stress remains to be investigated. Interestingly, deletion of *Crh* from GABAergic forebrain neurons confers resilience to chronic stress-induced anxiety, supporting the hypothesis that CRH action can switch from positive to negative in the presence of chronic, uncontrollable stress. In contrast to acute stressors, which can be highly beneficial by priming the brain toward optimal alertness, behavioral and cognitive performance, adverse life events such as trauma and/or chronic stress represent strong risk factors for multiple neuropsychiatric disorders and can exacerbate mood-related psychopathologies. Thus, understanding the underlying molecular mechanisms by which specific stress circuits lose their motivational properties to become pathological is of utter importance for the development of novel treatments for stress-related psychiatric disorders.

## Data Availability

All datasets generated for this study are included in the manuscript and/or the supplementary files.

## Ethics Statement

The animal study was reviewed and approved by the Regierung von Oberbayern, Munich.

## Author Contributions

ND and JD conceived and designed the study, and wrote the manuscript. ND performed the experiments and analyzed the data. CK generated the conditional knockout mutants. CK, KG, and JH assisted with behavioral experiments and data acquisition. KR and MS contributed to the interpretation of the data and revised the manuscript.

## Conflict of Interest Statement

The authors declare that the research was conducted in the absence of any commercial or financial relationships that could be construed as a potential conflict of interest.
